# Access to cochlear implantation

**DOI:** 10.1179/1467010013Z.00000000081

**Published:** 2013-03

**Authors:** Donna L. Sorkin

**Affiliations:** Executive Director, American Cochlear Implant Alliance, Virginia, USA

This Supplement to *Cochlear Implants International* brings together the experiences and perspectives of cochlear implant (CI) clinicians, educators, and advocates from around the globe. Each of the authors has examined the issue of access in his or her country in an effort to explain why a great number of children and adults who could benefit from CIs are not receiving the intervention. While there is a good bit of diversity relative to CI availability in the five countries reviewed (Belgium, China, Japan, the UK, and the USA), there are also some important commonalities.

There is considerable variation in CI utilization by appropriate children and adults in the countries represented. In the Flanders area of Belgium, pediatric utilization rates are estimated at 93% of eligible children, with the UK and some European countries also reaching over 90% if the age at the time of implant is extended into the mid-teen years. In stark contrast, about 50% of US children who could benefit are receiving CI and the utilization rate is lower still in Japan. In China, where cochlear implantation was later to begin on a large scale, the country's huge population and dramatic income disparities combine to keep China's current utilization rate relatively low though this could change in the future with current CI growth at 25% per year.

Adding adults into the mix changes the utilization dynamic dramatically. None of the countries represented in our interesting (though unscientific) sample of five are reaching anywhere close to the number of adults who theoretically could benefit from cochlear implantation. Although adult recipient numbers are growing, it has been estimated that no more than 5% of candidate adults in the USA receive CI and the rate is likely to be even lower in the European and Asian countries represented in this issue.


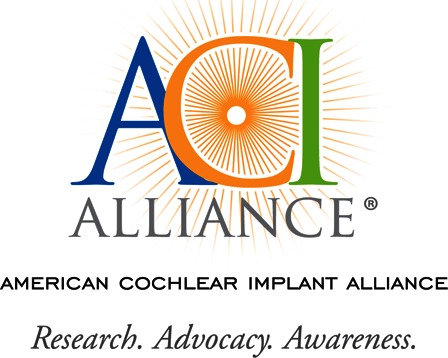


The reasons for this underutilization include: (1) low awareness of the benefits of CIs among the general population and among health care professionals; (2) the lack of specific referral pathways; (3) political issues relating to the deaf community, particularly in the USA; (4) financial issues related to provision of CI whether from private insurers (USA) or from government entities; and (5) relatively stringent candidacy requirements (especially in Japan). In many instances, the above problems could be mitigated to some degree if there were clearly articulated and adopted clinical practice guidelines to provide guidance to all the individuals and governmental entities that influence access to cochlear implantation. In the USA some of these issues could be improved by national agreement for best clinical practice in respect of CI access, the general acknowledgement of cost-effectiveness data, and the presence of an organization dedicated to the subject of cochlear implantation. Such an organization, the American Cochlear Implant Alliance, is described in the paper by Niparko *et al.* in this Supplement.

It is our hope that the thoughtful articles that comprise this supplement will help advance discussions and encourage solutions so that as many eligible children and adults as possible may have full access to the life-changing benefits provided by cochlear implantation.

